# All-Cause Mortality and Cancer Risk Dependent on Blood Se Level and HRG rs10770 Genotypes on a Prospective Cohort of Women with Familial Breast Cancers

**DOI:** 10.3390/ijms27052402

**Published:** 2026-03-05

**Authors:** Krzysztof Lubiński, Adam Stachowski, Wojciech Marciniak, Róża Derkacz, Adam Kiljańczyk, Milena Kiljańczyk, Marcin R. Lener, Sandra Pietrzak, Cezary Cybulski, Tadeusz Dębniak, Tomasz Huzarski, Wojciech Kluźniak, Tadeusz Sulikowski, Jan Lubiński, Rodney J. Scott, Jacek Gronwald

**Affiliations:** 1 International Hereditary Cancer Center, Department of Genetics and Pathology, Pomeranian Medical University in Szczecin, ul. Unii Lubelskiej 1, 71-252 Szczecin, Poland; krzychul123456789@gmail.com (K.L.); adam.stachowski@pum.edu.pl (A.S.); adam.kiljanczyk@pum.edu.pl (A.K.); milena.matuszczak@pum.edu.pl (M.K.); marcinlener@poczta.onet.pl (M.R.L.); sandra.pietrzak@pum.edu.pl (S.P.); cezarycy@pum.edu.pl (C.C.); debniak@pum.edu.pl (T.D.); huzarski@pum.edu.pl (T.H.); wojciech.kluzniak@pum.edu.pl (W.K.); lubinski@pum.edu.pl (J.L.); 2 Read-Gene, Grzepnica, ul. Alabastrowa 8, Grzepnica, 72-003 Dobra (Szczecińska), Poland; wojciech.marciniak@read-gene.com (W.M.); roza.derkacz@gmail.com (R.D.); 3 Department of Diagnostic Imaging and Interventional Radiology, Pomeranian Medical University Hospital No 1, 70-204 Szczecin, Poland; 4 Department of Clinical Genetics and Pathology, University of Zielona Góra, ul. Zyty 28, 65-046 Zielona Góra, Poland; 5 Department of General, Minimally Invasive, and Gastroenterological Surgery, Pomeranian Medical University in Szczecin, 71-252 Szczecin, Poland; tadeusz.sulikowski@pum.edu.pl; 6 Priority Research Centre for Cancer Research, Innovation and Translation, Hunter Medical Research Institute, New Lambton Heights, NSW 2308, Australia; rodney.scott@newcastle.edu.au; 7 School of Biomedical Sciences and Pharmacy, Faculty of Health and Medicine, University of Newcastle, Callaghan, NSW 2308, Australia; 8 Division of Molecular Medicine, Pathology North, John Hunter Hospital, New Lambton, NSW 2305, Australia

**Keywords:** Se levels, cancer risk, prospective study, all-cause mortality, *HRG* rs10770, familial breast cancers

## Abstract

The aim of this study was to investigate whether genotypes of *HRG* may modify the effect of Se(selenium) on all-cause mortality and cancer risk. The study was conducted on 2782 initially unaffected women from families with familial breast cancers, all registered at the Hereditary Cancer Centre in Szczecin. Participants were aged 40 years or older and were recruited between September 2010 and March 2024. Women carrying a BRCA1 mutation and those with a diagnosed cancer were excluded from the study. Blood Se levels were measured using inductively coupled plasma mass spectrometry, and molecular analyses of *HRG* (Histidine-rich glycoprotein) genotypes were performed using real-time PCR with TaqMan probes. After an average follow-up period of 6 years and 2 months, 89 deaths and 210 cases of cancer were identified. The study showed significant differences in the reference range of blood selenium levels, as well as its impact on all-cause mortality and cancer risk, depending on *HRG* genotype. The most striking finding regarding all-cause mortality risk was observed among women over 50 years of age, effect estimates are presented as hazard ratios (HRs) with 95% confidence intervals (CIs). Regardless of genotype, women with blood selenium (Se) levels in the lowest quartile (Q1) had a significantly higher all-cause mortality risk compared with those in the highest quartile (Q4), (HR = 3.07; 95%CI: 1.53–6.16; *p* = 0.001). Among women with the *HRG* non-TT genotype, the risk was even more pronounced—those with Se levels in Q1 had a significantly increased mortality risk compared with women in the higher quartiles (Q2–Q4) (HR = 7.68; 95%CI: 2.31–25.47; *p* = 0.0008). In contrast, for carriers of the *HRG* TT genotype, increased all-cause mortality risk was observed only when blood Se levels were in Q1 compared with Q4 (HR = 2.40; 95%CI: 1.09–5.31; *p* = 0.029). Important findings for any cancer risk have emerged in women below 50 years of age. Among women with the *HRG* TT genotype, the cancer risk was significantly increased in Q1 compared with women in Q2 (HR = 4.15; 95%CI: 1.55–11.06; *p* = 0.004). In contrast, the results for *HRG* non-TT carriers, regardless of genotype, were statistically insignificant. In summary, mortality and cancer risk appeared to be dependent on *HRG* genotype and blood Se levels.

## 1. Introduction

Familial breast cancers represent approximately 30% of all diagnosed breast cancers. An estimated 3–4% of women meet the clinical criteria for hereditary breast cancer, placing them at a substantially elevated risk of developing malignancy and all-cause mortality up to a fourfold higher risk compared with the general population. Beyond inherited predispositions, it is also essential to identify additional factors—both genetic and environmental- that may further modify cancer risk [1,2].

Selenium is an essential trace element incorporated into a diverse family of selenoproteins, which function in antioxidant defense, redox regulation, immune activity, and thyroid hormone metabolism. Through these mechanisms, selenium contributes to cellular protection against oxidative stress and chronic inflammation, two key processes associated with tumour development. Recent discoveries of disease-associated polymorphisms in selenoprotein genes further highlight inter-individual variation in selenium metabolism and its potential influence on cancer susceptibility [3,4].

Low selenium status has been associated with higher all-cause mortality, increased cardiovascular mortality, impaired immune function, and faster cognitive decline. Observational evidence indicates that higher selenium concentrations—particularly when measured through internal biological biomarkers such as serum, plasma or whole blood—are linked to a lower incidence and cancer mortality from several major malignancies. Prospective studies consistently demonstrate inverse associations between circulating selenium levels and the risk of prostate, lung, colorectal, bladder, gastric, hepatic, and pancreatic cancers, as well as reductions in total cancer incidence and all-cause mortality. For example, dose–response analyses show that every 10 μg/L increase in serum selenium corresponds to a ~26% reduction in overall cancer incidence and a ~6% decrease in cancer mortality [5,6,7,8,9,10].

Evidence suggests that the relationship between selenium and cancer follows a U-shaped or a nonlinear pattern, where both low and high selenium levels are associated with increased cancer incidence and mortality, as well as all-cause mortality, while intermediate levels confer the greatest protection. Such nonlinear relationships have been reported for overall cancer risk, cancer-specific outcomes, and several individual tumor sites, demonstrating that selenium exerts optimal effects only within a narrow physiological range [11].

Similar relationships have been reported. Large epidemiological studies have revealed U-shaped associations, in which both low and high selenium intake increase cancer risk. This was exemplified in a major study from Vietnam, where both low Se intake (27.8–77.2 µg/day) and high intake (169.1–331.7 µg/day) were associated with markedly increased risk of cancers of the stomach, colon, rectum, and lung, whereas the lowest risk was observed at intermediate intakes (110–124 µg/day). Similarly, for melanoma, toenail selenium showed an inverted U-shaped pattern, with an elevated risk at intermediate levels and lower risk at both low and high concentrations, reflecting heterogeneity across study designs [12].

Collectively, available evidence portrays selenium as a nutrient with a narrow optimal range, where inadequate levels increase the risk of several cancers and cancer-related mortality, but excessive intake may also elevate the risk of specific malignancies and metabolic disorders. Understanding these U-shaped, J-shaped, and inverse relationships and identifying the biological and population-level thresholds at which selenium shifts from protective to potentially harmful is essential for developing informed public health recommendations and for interpreting selenium’s role in cancer prevention or progression [3,4,5,6,7,8,9,10,11,12].

Given the substantial heterogeneity reported in previous findings, including inverse, U-shaped, and J-shaped associations, as well as discrepancies between observational studies and randomized trials, the role of selenium in cancer development and cancer-related mortality remains incompletely understood. Moreover, most available data originate from non-Central European populations, leaving significant uncertainty regarding whether these associations apply to countries with distinct selenium status profiles, such as Poland, where environmental selenium availability is relatively low. In addition, growing evidence suggests that genetic variation in selenoprotein-related pathways may modify the biological effects of selenium.

At our centre, we have performed an extensive search for gene candidates that potentially influence the relationship between selenium and cancer incidence/cancer mortality and all-cause mortality. Through this approach, *HRG* was selected as a promising candidate to modify the Se reference ranges associated with cancer incidence and all-cause mortality for women from familial breast cancer families. From our pilot study, we had some evidence that polymorphisms in *HRG* were associated with cancer incidence/all-cause mortality in a cohort of 329 unaffected women. These results were comparable to data from another cohort in which we maintained Se(selenium) levels at an optimal level to reduce cancer risk and mortality and found that polymorphisms in *HRG* significantly influenced the risk of death depending on Se levels.

## 2. Results and Discussion

**Figure 1:**
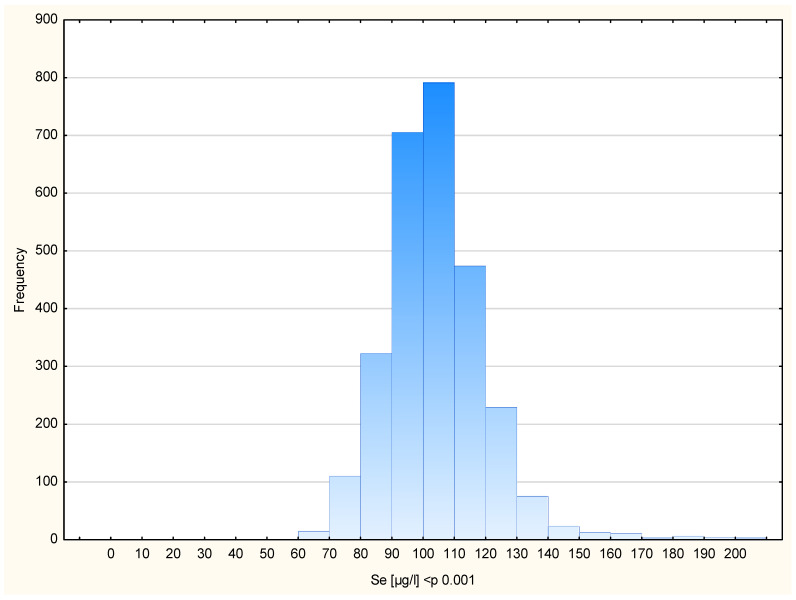
The distribution of the blood Se levels in all patients.

The final study cohort comprised 2782 women free of cancer at baseline. Each participant provided a single blood sample used for Se measurement. The mean age at the time of sample collection was 52.4 years (range: 40–83 years). Participant characteristics are summarized in Table 1. The average blood Se concentration in the entire cohort was 104.43 µg/L, with mean values by subgroup also presented in Table 1. Blood Se concentrations are shown in Figure 1, which illustrates the distribution of blood Se levels in the entire study cohort.

The mean follow-up duration was 6.2 years (range: 0.5–13.5 years). During this period, 210 cancer cases were identified, including 106 breast cancers and 104 malignancies of other types (Table 2). The causes of death in the cohort are presented in (Table 3. Among the 82 recorded deaths during the follow-up period, 44 were cancer-related, 30 were due to non-cancer causes, and 8 were from unknown causes.

The 2782 cancer-free women were divided into four equally sized groups (quartiles) based on their total blood Se concentrations. The quartile cut-off values were defined as follows: Q1 < 93.96 µg/L, Q2 = 93.96–102.81 µg/L, Q3 = 102.81–112.40 µg/L, and Q4 > 112.40 µg/L. Analyses of cancer risk and death risk were performed for the entire cohort and for subgroups stratified by age (<50 and ≥50 years) and cancer type (any cancer and breast cancer).

### 2.1. The Whole Group

#### 2.1.1. The Whole Group-Any Cancer Risk

The results for any cancer risk in the whole group without genotyping reveal a slight but statistically significant difference between the first and second Se quartiles, Q1 vs. Q2, where any cancer risk was lower in Q2 [Table S1]. In women with *HRG* TT genotype, the difference between Q1 and Q2 became more pronounced compared with the previous analysis [Table 4]. The results for women with *HRG* non-TT were not statistically significant [Table S2].

#### 2.1.2. Total Group Risk of Death

Results for mortality risk in the entire group without stratifying by genotype revealed that the lowest mortality risk was observed in Q4, with a statistically significant difference compared to Q1 [Table 5].

**Figure 2:**
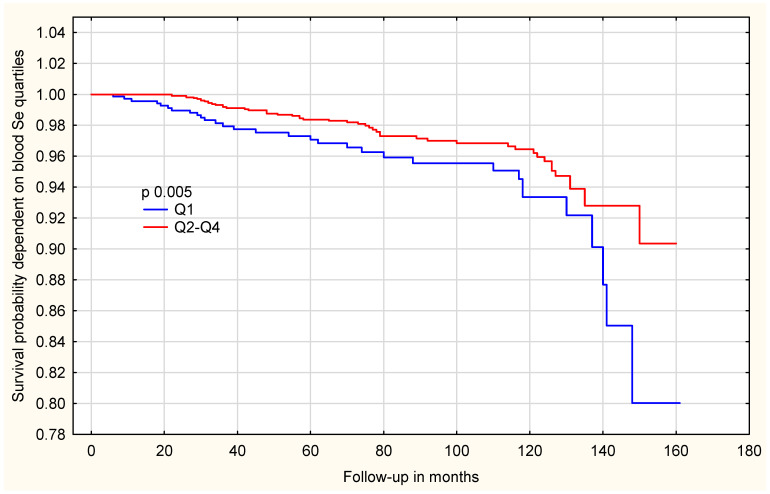
Survival probability, dependent on blood Se level in the whole group, regardless of genotype.

**Figure 3:**
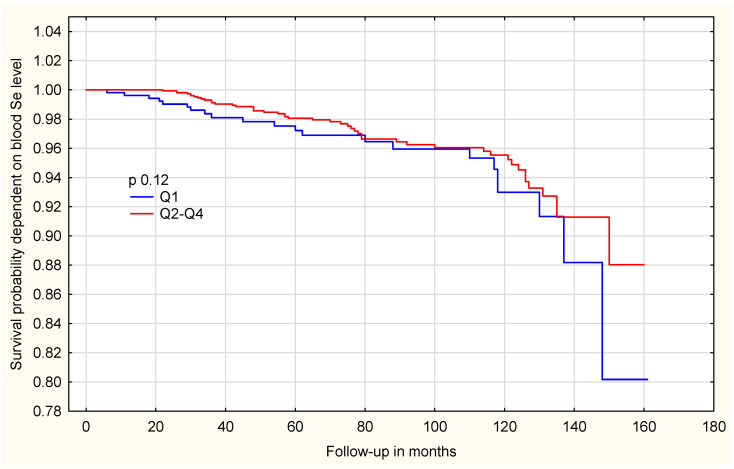
Survival probability, dependent on blood Se level in the whole group of patients with *HRG* TT genotype.

Following genotype adjustment, the observed difference was not significant among women with the *HRG* TT genotype [Table S3]. In contrast, among women with the *HRG* non-TT genotype, the association was markedly stronger, effect estimates are presented as hazard ratios (HRs) with 95% confidence intervals (CIs). Those in the lowest selenium quartile (Q1) had a significantly higher all-cause mortality risk compared to women in the three higher quartiles (Q2–Q4) (HR = 7.93; 95%CI: 2.39–26.26; *p* = 0.0006) [Table 6]. The differences in overall survival between selenium quartiles are shown in Figure 2. Women in the lowest selenium quartile (Q1) showed a clearly reduced survival probability compared with those in the higher quartiles (Q2–Q4). To further explore whether this association differs by genotype, survival analyses were stratified by *HRG* genotype. Among women with the *HRG* TT genotype, the survival curves are shown in Figure 3.

**Figure 4:**
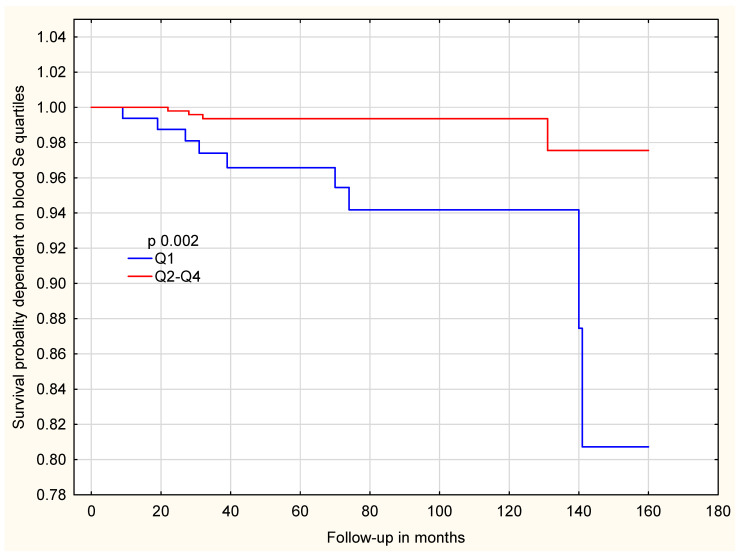
Survival probability, dependent on blood Se level in the whole group of patients with *HRG* non-TT genotype.

In contrast, among women with the *HRG* non-TT genotype, the association between selenium levels and survival was much stronger. As shown in Figure 4, women in the lowest selenium quartile (Q1) had significantly reduced survival compared with those in Q2–Q4 (*p* = 0.002).

### 2.2. The Subgroup Aged Above 50 Years

#### 2.2.1. Women Above 50 Years of Age: Any Cancer Risk

Among women aged over 50 years, no statistically significant association was found between selenium levels and cancer risk [Table S4]. This result remained non-significant after accounting for *HRG* TT [Table S5] and non-TT [Table S6] genotypes.

#### 2.2.2. Women Above 50 Years of Age and the Risk of Death

In women aged over 50 years, in the absence of genotyping, a significant association between Se levels and mortality risk was observed, with the lowest risk in Q4 compared with Q1 (HR = 3.07; 95%CI: 1.53–6.16; *p* = 0.001) [Table 7].

When taking into consideration the *HRG* TT genotype, the association remained statistically significant, even though the effect was weaker than in the unstratified analysis [Table S7]. Among women aged over 50 years carrying the *HRG* non-TT genotype, a strong and statistically significant association was observed. Those with selenium levels in the lowest quartile (Q1) had a markedly higher mortality risk compared to women in the higher quartile groups (Q2–Q4) (HR = 5.92; 95%CI: 1.26–27.68; *p* = 0.02) [Table 8].

### 2.3. The Subgroup at Age Below 50

#### 2.3.1. The Subgroup at Age Below 50-Any Cancer Risk

Among women aged below 50 years, without consideration of genotype, no statistically significant association was observed between Se levels and cancer risk [Table S8]. For *HRG* TT genotype carriers, a strong and statistically significant association was observed. The lowest cancer risk occurred in the second Se quartile (Q2), in contrast to the highest risk observed in Q1 (HR = 4.15; 95%CI: 1.55–11.06; *p* = 0.004) [Table 9]. In contrast, the association was not significant among women with the *HRG* non-TT genotype [Table S9].

#### 2.3.2. The Subgroup of Women Below 50 Years of Age and the Risk of Death

Among women under 50 years of age, their mortality risk showed no statistically significant association with Se levels, either in the overall analysis [Table S10] or after stratification by *HRG* genotype (Table S11 and non-TT Table S12).

Selenium is a well-established micronutrient that plays an important role in human health, with numerous studies linking it to both cancer risk and all-cause mortality [5,6,7,8,9,10,13]. In our study, we identified *HRG* as a significant genetic factor that modified the effect of selenium both on cancer risk and all-cause mortality.

Selenium appears to play a more important role in determining mortality risk than in cancer incidence, and its effect on mortality seems to be strongly influenced by *HRG* genotype.

Among the analyzed genotypes, the *HRG* non-TT genotype showed the strongest and statistically significant association with mortality risk. In the present cohort, women from breast cancer families carrying this genotype who had selenium blood levels above 93.96 µg/L (above the first quartile) exhibited a lower observed risk of all-cause mortality compared with those with lower selenium concentrations. This pattern was consistently observed across the analyzed subgroups, including women younger than 50 years of age.

In women under 50 years of age, the effect of the *HRG* non-TT genotype could not be formally evaluated due to the absence of deaths in this subgroup. Nevertheless, within the limits of the available data, selenium levels above 93.96 µg/L were not associated with adverse outcomes, suggesting that such concentrations may represent a non-harmful or potentially protective range in this population.

These findings should be interpreted with caution, as they are based on a single prospective cohort and do not constitute clinical recommendations. Rather, they indicate a potential gene–environment interaction between *HRG* genotype and selenium status, which warrants confirmation in independent cohorts and interventional studies.

While the *HRG* TT genotype showed no significant association with mortality, it appeared relevant to cancer risk, particularly in women younger than 50 years of age. In this subgroup, selenium blood concentrations within the range of 93.96–102.81 µg/L (second quartile) were associated with the lowest observed cancer incidence.

Importantly, the identified selenium threshold should not be interpreted as an optimal or target concentration, but rather as a data-driven cut-point used to illustrate differences in risk within this cohort.

It is important to note that genotyping allows the identification of high-risk groups of women with a markedly increased probability of death and risk of developing cancer. By incorporating genetic information, it may be possible to identify subgroups in which selenium status is differentially associated with cancer incidence and mortality risk. Moreover, the results indicate a stronger influence of blood selenium levels on cancer and mortality outcomes.

The strength of this study lies in the high hazard ratios, strong statistical significance, and narrow confidence intervals. Since some of the presented results are based on a relatively small number of events, despite achieving statistical significance, they can be unstable and should be interpreted with caution. Absence of deaths among *HRG* non-TT carriers younger than 50 years of age represents an important limitation, as it precluded formal estimation of hazard ratios in this subgroup and limits the generalizability of the findings to younger women.

The selection of *HRG* polymorphisms appears to have been appropriate; however, it is worth noting that two other gene polymorphisms (*APOB* rs1367117 and *MKI67* rs11016073) were assessed, but neither was associated with any difference in cancer risk or mortality. Only *HRG* showed statistically significant results. This does not rule out other selenoproteins, and further analyses are planned for future studies.

*HRG* has been linked to pathways involved in cell proliferation and survival, including DNA replication and cell cycle regulation, through interactions with genes such as *MCM7*, *PCNA*, *CDK6*, and *CCNB1*. Selenium, as an essential component of selenoproteins involved in antioxidant defense, may indirectly modulate these pathways by limiting oxidative stress and maintaining redox balance, which are critical for proper cell cycle control.

One possible explanation is that *HRG*-related regulation of proliferative pathways may alter redox homeostasis, thereby influencing the selenium levels required for optimal antioxidant defense. In this context, different *HRG* genotypes may be associated with distinct selenium ranges that are sufficient to counterbalance oxidative stress and dysregulated proliferation.

Although *HRG* has been linked to genes involved in selenium metabolism, the precise molecular mechanisms underlying this interaction remain unclear and warrant further functional investigation [14,15].

The relevance of *HRG* to the present findings lies in its role as a regulator of angiogenesis, immune responses, and tissue remodeling—processes that are highly sensitive to oxidative stress and redox balance.

Many of the biological processes regulated by *HRG*—including angiogenesis, macrophage polarization, and extracellular matrix remodeling—are strongly influenced by oxidative stress and inflammatory signaling. Selenium-dependent antioxidant systems, particularly selenoproteins involved in redox regulation, may therefore modulate the functional consequences of *HRG* activity.

We hypothesize that genetic variation in *HRG* may alter the efficiency or context in which these redox-sensitive pathways operate, thereby modifying the selenium levels required to maintain cellular homeostasis and limit tumor-promoting processes.

HRG (Histidine rich glycoprotein) is a liver-derived plasma protein that regulates angiogenesis, immune responses, and tissue remodeling through interactions with multiple ligands, including heme, thrombospondin-1, and components of the complement and coagulation systems. Experimental models indicate that HRGplays a context-dependent role in cancer biology, influencing macrophage polarization, fibrosis, angiogenesis, and metastatic potential. Dysregulation of *HRG* expression—either increased or decreased—has been associated with adverse outcomes in cancer and liver-related pathologies, underscoring its role in redox- and inflammation-sensitive pathways [16,17,18,19,20,21].

### 2.4. Limitations

The potential diagnostic relevance of our findings is considerable; however, validation in independent cohorts, including both Polish and other ethnic groups, is required. Although data on occupational exposure were collected, socio-economic status was not evaluated. Furthermore, blood Se concentration was determined from a single sample obtained at baseline, which may not fully reflect long-term exposure.

## 3. Materials and Methods

### 3.1. Study Group

Recruited women aged 40 years and above were examined at the Cancer Genetics Outpatient Clinic and deemed to be unaffected by any cancer. Each participant underwent screening for at least three founder *BRCA1* mutations that are most prevalent in the Polish population (c.5266dupC–5382insC, c.181T>G–300T>G, and c.4035delA–4153delA). Collectively, these variants account for over 90% of hereditary breast cancer cases related to *BRCA1* mutations in Poland [22].

Genetic testing was performed at the Pomeranian Medical University in Szczecin between September 2010 and March 2024. None of the enrolled women had a previous cancer diagnosis at baseline. Written informed consent was obtained from all participants, including permission for blood collection for scientific purposes. Routine follow-up assessments were carried out annually.

The study protocol was approved by the Ethics Committee of the Pomeranian Medical University in Szczecin and complied with the principles of the Declaration of Helsinki. During the initial clinic visit, a blood specimen was taken for genetic evaluation. An additional 10 mL whole-blood sample was collected and preserved at −80 °C for potential future analyses.

Participants also completed a structured questionnaire covering demographic and clinical characteristics such as family and personal history of cancer, smoking habits, hormone use, and previous surgeries, including oophorectomy.

### 3.2. Measurement of Blood Se

Venous blood was drawn from fasting participants using the Vacutainer^®^ System (cat. no. 368381, Becton Dickinson, Plymouth, UK). Samples were divided into cryovials and stored at −80 °C until further use.

Elemental determinations were carried out by inductively coupled plasma mass spectrometry (ICP-MS; NexION 350D, PerkinElmer, Norfolk, VA, USA) operated in Kinetic Energy Discrimination (KED) mode. Rhodium served as the internal standard to correct for instrumental fluctuations and potential matrix interferences. Detailed analytical settings of the instrument are available upon request.

Before analysis, specimens were diluted 1:40 (70 µL of blood with 2730 µL of diluent). The diluent contained ultrapure water (>18 MΩ), tetramethylammonium hydroxide (TMAH; AlfaAesar, Kandel, Germany), Triton X-100 (PerkinElmer, Shelton, CT, USA), ethylenediaminetetraacetic acid (EDTA; Merck, Darmstadt, Germany), and ethanol (Merck). Calibration standards were prepared from a 1000 µg/mL iodine stock solution (PerkinElmer Pure Plus, Shelton, CT, USA) diluted with the same matrix. The calibration approach was matrix-matched, and all calibration curves showed correlation coefficients (R^2^) exceeding 0.999.

Measurement accuracy and repeatability were verified using certified reference materials: ClinChek^®^ Plasmonorm Whole Blood Level 1 (Recipe, Munich, Germany) and Seronorm Whole Blood Level 2 (Sero, Norway). The laboratory participates in the external proficiency testing program QMEQAS (Québec Multielement External Quality Assessment Scheme), coordinated by the Institut National de Santé Publique du Québec. Additional details regarding plasma operating conditions and acquisition parameters are available upon request.

#### Quality Control

To ensure analytical accuracy and reproducibility, all determinations were validated using certified reference material (CRM)—ClinChek^®^ Plasmonorm Whole Blood Trace Elements Level 1 (Recipe, Munich, Germany). Recovery rates ranged from 80% to 105%, and the coefficient of variation (CV%) remained below 15%.

The analytical laboratory participates in two independent external proficiency testing programs: the Lead and Multielement Proficiency (LAMP) program coordinated by the U.S. Centers for Disease Control and Prevention (CDC), and the Québec Multielement External Quality Assessment Scheme (QMEQAS) supervised by the Institut National de Santé Publique du Québec.

### 3.3. Genotype Assessment

Genotyping was carried out using a real-time polymerase chain reaction (PCR) on a LightCycler 480 system (Roche Diagnostics, Mannheim, Germany). The reactions employed TaqMan chemistry with predesigned assay mixtures containing sequence-specific primers and fluorescent probes (Applied Biosystems, Foster City, CA, USA). For the *HRG* rs10770 variant, a custom assay was developed. Each reaction was performed at a total volume of 5 μL. The reaction mixture consisted of 2.5 µL of 2× Probe qPCR Master Mix (Promega Corporation, Madison, WI, USA), 0.125 µL of the 40× TaqMan Assay (Applied Biosystems), 1.375 µL of nuclease-free water, and 1 µL of genomic DNA (25 ng/µL). Real-time PCR was carried out on a LightCycler 480 instrument (Roche).

The cycling protocol started with an initial denaturation at 95 °C for 10 min, followed by 50–55 amplification cycles of 95 °C for 10 s, 52 °C for 30 s (annealing), and 72 °C for 10 s (extension).

After amplification, the run concluded with a cooling step and instrument-specific color-compensation procedures according to the manufacturer’s recommendations (40 °C for 30 s, followed by brief denaturation and hybridization at 80 °C). Fluorescence was monitored continuously during the hybridization phase, with 1 °C temperature increments applied when required.

### 3.4. Genotype Selection

To identify genes potentially interacting with Se and influencing cancer susceptibility and/or mortality, a comprehensive PubMed literature search was conducted. Genes were considered for inclusion if they showed any direct or indirect interaction with Se and had previously been implicated in carcinogenesis or mortality. This initial screening yielded 190 candidate genes.

In the second stage, polymorphisms within these genes were investigated using the Polinome Genomic Variants Database, which contains data from 300 healthy Polish adults from cancer-free families analyzed by whole-exome sequencing. Variants were selected based on prior reports or bioinformatic predictions suggesting a functional effect on protein structure or activity. Additionally, only polymorphisms with genotype frequencies between 25% and 75% were considered.

Following this filtering process, five genes met the inclusion criteria: *CASP9* rs2234723, *GPX1* rs1050450, *APOB* rs1367117, *MKI67* rs11016073, and *HRG* rs10770.

Of these five genes, three polymorphisms in *HRG* rs10770, *MKI67* rs11016073, *APOB* rs1367117 were prioritized for the present study based on findings from an independent case–control cohort (n = 329; ratio 1:2) derived from a larger prospective group of 2927 women from hereditary breast cancer families registered at the Hereditary Cancer Center in Szczecin. 

### 3.5. Statistical Analysis

Information on cancer incidence was obtained from hospital medical records and pathology reports. Participants were divided into four equal quartiles according to blood Se concentration among cancer-free women, ensuring an even distribution across groups. Quartile cutoffs for Se levels, derived from the entire study cohort, were used in subsequent analyses.

Follow-up began at the time of blood collection or at age 40—whichever occurred later—and continued until the diagnosis of the first cancer, death from another cause, or August 2024, whichever came first.

Associations between Se exposure, all-cause mortality and cancer risk were assessed using multivariate Cox proportional hazards models to estimate hazard ratios (HRs) and 95% confidence intervals (CIs). Models were adjusted for age at blood draw (<50 vs. ≥50 years), smoking status, family history of cancer in first-degree relatives, oophorectomy, oral contraceptive use, and hormone replacement therapy. All statistical analyses were performed using STATISTICA software (version 13.3, TIBCO Software Inc., Palo Alto, CA, USA).

Selenium levels were transformed into categorical variables by dividing the data sorter in ascending order into four equally sized categories (quartiles—Q1–Q4). Quartiles were used as cut-off thresholds. In each regression model, the reference category was defined as the quartile with the lowest proportion of events in relation to the total number of individuals. This approach aimed to avoid HR values below one (1.0), improving the interpretability of HRs between models. Due to the low frequency of events within individual quartiles, we decided combine quarters in such cases to enhance the stability of the results.

## 4. Conclusions

In summary, optimal blood selenium levels can be determined once genotyping is performed. Therefore, further studies are warranted to validate and extend these findings. Taken together with blood selenium concentration, DNA variants may serve as valuable biomarkers for assessing cancer susceptibility and mortality risk.

## Figures and Tables

**Table 1:** Characteristics of 2782 women in the cohort.

Characteristics	Death	Alive	Univariate Risk of Death HR; (95%CI); *p*	Multivariate Risk of Death HR; (95%CI); *p*	Cases	Unaffected	Univariate Cancer Risk HR; (95%CI); *p*	Multivariate Cancer Risk HR; (95%CI); *p*	Mean Se Level 104.43 µg/L, SD 21.60
Blood Se Levels by quartiles µg/L									
Q1 < 93.96	31 (4.4%)	665 (95.6%)	HR 2.46; 95%CI (1.26–4.80); *p* = 0.008	HR 2.71; 95%CI (1.38–5.31); *p* = 0.003	65 (9.3%)	631 (90.7%)	HR 1.53; 95%CI (1.04–2.26); *p* = 0.028	HR 1.55; 95%CI (1.059–2.29); *p* = 0.024	86.26 ± 6.382
Q2 93.96–102.81	19 (2.7%)	676 (97.3%)	HR 1.43; 95%CI (0.69–2.95); *p* = 0.33	HR 1.53; 95%CI (0.74–3.16); *p* = 0.25	43 (6.2%)	652 (93.8%)			98.52 ± 2.54
Q3 102.81–112.40	20 (2.8%)	675 (96.3%)	HR 1.64; 95%CI (0.80–3.59); *p* = 0.17	HR 1.65; 95%CI (0.80–3.40); *p* = 0.16	49 (7%)	646 (93%)	HR 1.17; 95%CI (0.78–1.77); *p* = 0.43	HR 1.14; 95%CI (0.75–1.72); *p* = 0.52	107.19 ± 2.79
Q4 > 112.40	12 (1.7%)	684 (98.3%)			53 (7.6%)	643 (92.4%)	HR 1.29; 95%CI (0.86–1.93); *p* = 0.20	HR 1.20; 95%CI (0.80–1.79); *p* = 0.37	112.40 ± 31.36
Genotype									
*HRG* rs10770 TT	69 (3.2%)	2042 (96.8%)	HR 1.73; 95%CI (0.96–3.14); *p* = 0.06	HR 1.69; 95%CI (0.93–3.07); *p* = 0.08	162 (7.6%)	1949 (92.4%)	HR1.08; 95%CI (0.78–1.49); *p* = 0.63	HR1.0695%CI (0.77–1.47); *p* = 0.70	104.14 ± 16.39
*HRG* rs10770 non-TT	13 (1.9%)	658 (98.1%)			48 (7.1%)	623 (92.9%)			105.37 ± 33.00
Cancers in first-degree relatives									
No	12 (2.6%)	446 (97.4%)			34 (1.2%)	424 (15.2%)			105.56 ± 38.36
Yes	70 (3%)	2254 (97%)	HR 1.26; 95%CI (0.68–2.34); *p* = 0.44	HR 1.22; 95%CI (0.66–2.27); *p* = 0.52	176 (6.4%)	2148 (77.2%)	HR1.05; 95%CI (0.73–1.52); *p* = 0.78	HR 0.9995%CI (0.68–1.44); *p* = 0.98	104.21 ± 16.39
Oral Contraceptives									
No	74 (3.6%)	1969 (96.4%)			169 (6.1%)	1874 (67.3%)			105.40 ± 43.88
Yes	8 (1%)	731 (99%)	HR 0.42; 95%CI (0.20–0.88); *p* = 0.02	HR 0.72; 95%CI (0.34–1.55); *p* = 0.41	41 (1.5%)	698 (25.1%)	HR0.83; 95%CI (0.59–1.18); *p* = 0.31	HR1.07; 95%CI (0.74–1.55); *p* = 0.69	105.97 ± 18.87
Oophorectomy									
No	78 (2.9%)	2527 (97.1%)			193 (6.9%)	2412 (86.7%)			104.27 ± 21.77
Yes	4 (2.2%)	173 (97.8%)	HR 0.81; 95%CI (0.29–2.22); *p* = 0.68	HR 0.68; 95%CI (0.25–1.88); *p* = 0.46	17 (0.65%)	160 (5.75%)	HR1.34; 95%CI (0.81–2.21); *p* = 0.24	HR1.18; 95%CI (0.71–1.9621); *p* = 0.51	106.86 ± 18.80
Hormone replacement therapy									
No	66 (3%)	2121 (97%)			158 (5.6%)	2029 (72.9%)			103.79 ± 22.85
Yes	16 (2.6%)	579 (97.4%)	HR 0.87; 95%CI (0.50–1.50); *p* = 0.62	HR 0.71; 95%CI (0.40–1.24); *p* = 0.23	52 (1.9%)	543 (19.6%)	HR1.19; 95%CI (0.88–1.64); *p* = 0.24	HR1.03; 95%CI (0.74–1.43); *p* = 0.83	106.25 ± 16.40
Smoking status									
No	32 (2.1%)	1430 (97.9%)			107 (3.8%)	1355 (48.7%)			104.58 ± 16.89
Yes (former or current)	50 (3.7%)	1270 (96.3%)	HR 1.58; 95%CI (1.015–2.47); *p* = 0.042	HR 1.40; 95%CI (0.89–2.20); *p* = 0.13	103 (3.7%)	1217 (43.8%)	HR1.02; 95%CI (0.78–1.34); *p* = 0.83	HR 0.97; 95%CI (0.74–1.27); *p* = 0.85	104.27 ± 25.83
Age									
<50	10 (0.8%)	1176 (99%)			55 (1.9%)	1131 (40.7%)			102.44 ± 16.31
≥50	72 (4.5%)	1524 (95.5%)	HR 4.11; 95%CI (2.12–7.98) *p* = 0.00002	HR 4.16; 95%CI (2.06–8.38) *p* = 0.00006	155 (5.6%)	1441 (51.9%)	HR1.74; 95%CI (1.28–2.37); *p* = 0.0004	HR1.77; 95%CI (1.26–2.50); *p* = 0.001	105.92 ± 24.71

Abbreviations: HR, hazard ratio; CI, confidence interval; Se, selenium; SD, standard deviation.

**Table 2:** Incident cancers detected in the cohort.

Cancer Site	n	Cases (%)	Mean Se Level µg/L, SD
None	2572		104.53 ± 21.68
Any Cancer	210	100	103.32 ± 20.53
Breast	106	50.43	101.48 ± 17.79
Lung	10	4.7	109 ± 21.79
Uterus	12	5.7	101.93 ± 14.35
Leukemia	4	1.9	108.64 ± 25.03
Lymphoma	3	1.4	121.42 ± 7.63
Bladder	4	1.9	107.50 ± 22.49
Thyroid	10	4.8	102.10 ± 14.68
Ovarian	14	6.7	114.46 ± 49.09
Cervix	5	2.4	109.87 ± 14.24
Myeloma	1	0.47	128.48
Melanoma	5	2.4	102.23 ± 7.38
Liver	1	0.47	103.51
Stomach	5	2.3	99.69 ± 12.05
Skin	9	4.3	102.04 ± 15.61
Glioma	1	0.47	96.40
Chondroma	1	0.47	114.04
Colon	10	4.9	99.51 ± 15.28
Parotid Gland	1	0.47	108.88
Kidney	5	2.4	95.76 ± 6.83
Abdominal Cavity	1	0.47	94.94
Pancreas	2	0.95	88.91 ± 1.66

**Table 3:** Causes of death in the cohort.

Cause of Death	n	Deaths (%)	Mean Se Level µg/L, SD
All Deaths	82	100	101.69 ± 25.161
Cancer	44	53.6	101.89 ± 30.45
Breast	13	15.9	96.31 ± 11.98
Lung	6	7.5	96.33 ± 13.87
Ovarian	5	6.1	142.47 ± 77.28
Glioma	5	6.1	98.46 ± 8.68
Colon	3	3.6	85.60 ± 6.99
Stomach	3	3.6	93.59 ± 3.38
Leukemia	3	3.6	114.89 ± 26.84
Bladder	2	2.4	106.43 ± 12.27
Metastatic malignancy, primary site unknown	2	2.4	87.65 ± 10.31
Kidney	1	1.2	91.76
Uterus	1	1.2	86.44
Non-Cancer	30	36.4	101.64 ± 18.17
Stroke	5	6.1	91.60 ± 11.86
COVID	4	4.9	116.87 ± 23.98
Sepsis	4	4.9	105.58 ± 16.84
Aneurysm	4	4.9	111.20 ± 25.73
Heart attack	3	3.6	107.16 ± 15.68
Chronic respiratory failure	3	3.6	88.55 ± 11.75
Chronic circulatory insufficiency	2	2.4	86.63 ± 11.22
Anesthetic complication (during non-cancer surgery)	1	1.2	88.53
Alzheimer	1	1.2	92.33
Parkinson	1	1.2	113.81
Renal failure	1	1.2	111.44
Sudden death during sleep	1	1.2	90.011
Unknown cause of death	8	10	100.76 ± 16.13

**Table 4:** Hazard ratios for any cancer risk by blood Se level irrespective of age with *HRG* TT genotype (quartiles).

			Univariate COX Regression			Multivariate COX Regression *		
Blood Se Level µg/L	Cases	Unaffected	HR	95%CI	*p*	HR	95%CI	*p*
Q1 <93.96	54 (10.1%)	479 (89.9%)	2.01	1.26–3.19	0.003	2.06	1.29–3.27	0.002
Q2 ref 93.96–102.81	27 (5.3%)	483 (94.7%)						
Q3 102.81–112.40	40 (7.4%)	498 (92.6%)	1.48	0.91–2.42	0.11	1.42	0.87–2.33	0.15
Q4 >112.40	41 (7.7%)	489 (92.3%)	1.58	0.97–2.57	0.06	1.46	0.89–2.38	0.13

* Adjusted for age, smoking status, first-degree relatives, adnexectomy, oral contraception, and hormone replacement therapy.

**Table 5:** Hazard ratios for risk of death by blood Se level irrespective of age (quartiles).

			Univariate COX Regression			Multivariate COX Regression *		
Blood Se Level µg/L	Dead	Alive	HR	95%CI	*p*	HR	95%CI	*p*
Q1 <93.96	31 (4.4%)	665 (95.6%)	2.46	1.26–4.80	0.008	2.7	1.41–5.40	0.003
Q2 93.96–102.81	19 (2.7%)	676 (97.3%)	1.43	0.69–2.95	0.3	1.55	0.70–2.90	0.23
Q3 102.81–112.40	20 (2.8%)	675 (97.2%)	1.6	0.80–3.35	0.17	1.70	0.83–3.48	0.1
Q4 ref >112.40	12 (1.7%)	684 (98.3%)						

* Adjusted for age, smoking status, first-degree relatives, adnexectomy, oral contraception, and hormone replacement therapy.

**Table 6:** Hazard ratios for risk of death by blood Se level irrespective of age with *HRG* non-TT genotype (quartiles).

			Univariate COX Regression			Multivariate COX Regression *		
Blood Se Level µg/L	Dead	Alive	HR	95%CI	*p*	HR	95%CI	*p*
Q1 <93.96	9 (5.5%)	154 (94.5%)	4.60	1.05–65.82	0.04	5.00	1.07–23.41	0.04
Q2 ref 93.96–102.81	2 (1%)	183 (99%)						
Q3 102.81–112.40	0	157						
Q4 >112.40	2 (1.2%)	164 (98.8%)	1.036	0.14–7.37	0.97	0.80	0.11–5.70	0.82
Q2–4 vs. Q1	4 (0.88%) 9 (5.5%)	503 (90.12%) 154 (94.5%)	6.62	2.03–21.54	0.001	7.93	2.39–26.26	0.0006

* Adjusted for age, smoking status, first-degree relatives, adnexectomy, oral contraception, and hormone replacement therapy.

**Table 7:** Hazard ratios for risk of death by blood Se level above 50 years of age (quartiles).

			Univariate COX Regression			Multivariate COX Regression *		
Blood Se Level µg/L	Dead	Alive	HR	95%CI	*p*	HR	95%CI	*p*
Q1 <93.96	29 (7.8%)	341 (92.2%)	3.20	1.59–6.41	0.001	3.07	1.53–6.16	0.001
Q2 93.96–102.81	16 (4.4%)	342 (95.6%)	1.57	0.73–3.40	0.24	1.51	0.69–3.26	0.29
Q3 102.81–112.40	16 (3.9%)	386 (96.1%)	1.59	0.73–3.42	0.23	1.54	0.71–3.32	0.27
Q4 ref >112.40	11 (2.3%)	450 (97.7%)						

* Adjusted for smoking status, first-degree relatives, adnexectomy, oral contraception, and hormone replacement therapy.

**Table 8:** Women over 50 years of age and the hazard ratios for the risk of death associated with blood Se levels in participants carrying the *HRG* non-TT genotype (quartiles).

			Univariate COX Regression			Multivariate COX Regression *		
Blood Se Level µg/L	Dead	Alive	HR	95%CI	*p*	HR	95%CI	*p*
Q1 <93.96	9 (11.2%)	71 (88.8%)	6.52	1.40–30.22	0.016	5.92	1.26–27.68	0.02
Q2 93.96–102.81	2 (2.1%)	90 (97.9%)	1.30	0.18–21.86	0.79	1.21	0.17–8.67	0.84
Q3 102.81–112.40	0	87						
Q4 ref >112.40	2 (1.7%)	116 (98.3%)						
Q2–4 vs. Q1 >112.40	4 (1.3%) 9 (11.2%)	293 (98.4%) 71 (88.8%)	8.26	2.54–26.85	0.0004	7.68	2.31–25.47	0.0008

* Adjusted for smoking status, first-degree relatives, adnexectomy, oral contraception, and hormone replacement therapy.

**Table 9:** Hazard ratios for any cancer risk by blood Se level below 50 years of age with *HRG* TT genotype (quartiles).

			Univariate COX Regression			Multivariate COX Regression *		
Blood Se Level µg/L	Cases	Unaffected	HR	95%CI	*p*	HR	95%CI	*p*
Q1 <93.96	21 (8.6%)	222 (91.4%)	4.04	1.52–10.72	0.005	4.15	1.55–11.06	0.004
Q2 ref 93.96–102.81	5 (2%)	238 (98%)						
Q3 102.81–112.40	8 (3.6%)	211 (96.4%)	1.86	0.61–5.69	0.27	1.86	0.61–5.70	0.27
Q4 >112.40	9 (4.8%)	178 (95.2%)	2.45	0.82–7.33	0.10	2.38	0.79–7.15	0.12

* Adjusted for smoking status, first-degree relatives, adnexectomy, oral contraception, and hormone replacement therapy.

## Data Availability

Data is unavailable due to privacy and ethical restrictions, available only upon request.

## Supplementary Materials

The supporting information can be downloaded at https://www.mdpi.com/article/10.3390/ijms27052402/s1.

## Abbreviations

IARC—International Agency for Research on Cancer; ICP-MS—Inductively Coupled Plasma Mass Spectrometry; CRM—certified reference material; JRC—Joint Research Centre; LAMP—Pb And Multielement Proficiency Program; CDC—Center for Disease Control; QMEQAS—Quebec Multielement External Quality Assessment Scheme; HR—hazard ratio; CI- confidence interval; RR—relative risk; LOD—limit of detection; LOQ—limit of quantification; SD—standard deviation; RSD—relative standard deviation; QCS2—Quick-Change Socket; HRG- Histidine rich glycoprotein.
