# Supplementary material for: All-Cause Mortality and Cancer Risk Dependent on Blood Se Level and HRG rs10770 Genotypes on a Prospective Cohort of Women with Familial Breast Cancers
Source: Int J Mol Sci. 2026 Mar 5;27(5):2402. doi: 10.3390/ijms27052402 (PMC12985312; doi:10.3390/ijms27052402)
Supplement: Supplementary file 1 [file ijms-27-02402-s001.zip › ijms-4095627-supplementary.pdf]

Table S1. Hazard ratios for any cancer risk by blood Se level irrespective on age(quartiles).

			Univariate COX Regression			Multivariate COX Regression *		
**Blood Se level** μg/L	**Cases**	**Unaffected**	**HR**	**95% CI**	***p*-value**	**HR**	**95% CI**	***p*-value**
Q1 <93.96	65(9.3%)	631(90.7%)	1.53	1.046-2.26	0.028	1.55	1.059-2.29	0.024
Q2 ref 93.96-102.81	43(6.2%)	652(93.8%)						
Q3 102.81-112.40	49(7.0%)	646(93%)	1.17	0.78-1.77	0.43	1.14	0.75-1.72	0.52
Q4 >112.40	53(7.6%)	643(92.4%)	1.29	0.86-1.93	0.20	1.19	0.80-1.79	0.37

***Adjusted to (age, smoking , 1st degree relatives, adnexectomy, oral contraception, hormone replacement therapy)**

Table S2. Hazard ratios for any cancer risk by blood Se level irrespective on age with *HRG* nonTT genotype (quartiles).

			Univariate COX Regression			Multivariate COX Regression *		
**Blood Se level** μg/L	**Cases**	**Unaffected**	**HR**	**95% CI**	***p*-value**	**HR**	**95% CI**	***p*-value**
Q1 <93.96	11(6.7%)	152(93.3%)	1.14	0.47-2.76	0.76	1.25	0.51-3.05	0.61
Q2 93.96-102.81	16(8.6%)	169(91.4%)	1.57	0.69-3.57	0.27	1.58	0.69-3.61	0.27
Q3 ref 102.81-112.40	9(5.7%)	148(94.3%)						
Q4 >112.40	12(7.2%)	154(92.8%)	1.26	0.53-2.99	0.59	1.14	0.47-2.74	0.76

***Adjusted to (age, smoking , 1st degree relatives, adnexectomy, oral contraception, hormone replacement therapy)**

Table S3. Hazard ratios for risk of death by blood Se level irrespective on age with *HRG* TT genotype (quartiles).

			Univariate COX Regression			Multivariate COX Regression *		
**Blood Se level** µg/L	**Dead**	**Alive**	**HR**	**95% CI**	***p*-value**	**HR**	**95% CI**	***p*-value**
Q1 <93.96	22(4.1%)	511(95.9%)	2.06	0.97-4.36	0.05	2.18	1.025-4.63	0.042
Q2 93.96-102.81	17(3.3%)	493(96.7%)	1.49	0.68-3.26	0.31	1.57	0.71-3.46	0.25
Q3 102.81-112.40	20(3.7%)	518(96.3%)	1.91	0.89-4.08	0.095	1.89	0.88-4.06	0.10
Q4 ref >112.40	10(1.9%)	520(98.1%)						

***Adjusted to (age, smoking , 1st degree relatives, adnexectomy, oral contraception, hormone replacement therapy)**

Table S4. Hazard ratios for any cancer risk by blood Se level above 50 years of age (quartiles).

			Univariate COX Regression			Multivariate COX Regression *		
**Blood Se level** µg/L	**Cases**	**Unaffected**	**HR**	**95% CI**	***p*-value**	**HR**	**95% CI**	***p*-value**
Q1 <93.96	42(9.3%)	328(90.7%)	1.38	0.87-2.19	0.16	1.38	0.87-2.19	0.16
Q2 ref 93.96-102.81	32(6.2%)	327(93.8%)						
Q3 102.81-112.40	39(7.0%)	367(93%)	1.15	0.72-1.83	0.55	1.13	0.71-1.82	0.58
Q4 >112.40	42(7.6%)	419(92.4%)	1.13	0.71-1.79	0.60	1.10	0.69-1.75	0.66

***Adjusted to (smoking , 1st degree relatives, adnexectomy, oral contraception, hormone replacement therapy)**

Table S5. Hazard ratios for any cancer risk by blood Se level above 50 years of age with *HRG* TT genotype (quartiles).

			Univariate COX Regression			Multivariate COX Regression *		
**Blood Se level μg/L**	**Cases**	**Unaffected**	**HR**	**95% CI**	***p*-value**	**HR**	**95% CI**	***p*-value**
Q1 <93.96	33(11.3%)	257(88.7%)	1.55	0.90-2.66	0.11	1.56	0.91-2.69	0.10
Q2 ref 93.96-102.81	22(8.2%)	245(91.8%)						
Q3 102.81-112.40	32(10%)	287(90%)	1.33	0.77-2.29	0.30	1.28	0.74-2.22	0.37
Q4 >112.40	32(9.3%)	311(90.7%)	1.29	0.75-2.23	0.34	1.26	0.73-2.17	0.40

***Adjusted to (smoking , 1st degree relatives, adnexectomy, oral contraception, hormone replacement therapy)**

Table S6. Hazard ratios for any cancer risk by blood Se level above 50 years of age with *HRG* nonTT genotype (quartiles).

			Univariate COX Regression			Multivariate COX Regression *		
**Blood Se level μg/L**	**Cases**	**Unaffected**	**HR**	**95% CI**	***p*-value**	**HR**	**95% CI**	***p*-value**
Q1 <93.96	9(11.2%)	71(88.8%)	1.41	0.52-3.80	0.49	1.53	0.56-4.16	0.40
Q2 93.96-102.81	10(10.8%)	82(89.2%)	1.37	0.52-3.62	0.51	1.41	0.53-3.77	0.48
Q3 ref 102.81-112.40	7(8%)	80(92%)						
Q4 >112.40	10(8.5%)	108(91.5%)	1.061	0.40-2.79	0.90	1.11	0.41-2.96	0.83

***Adjusted to (smoking , 1st degree relatives, adnexectomy, oral contraception, hormone replacement therapy)**

Table S7. Hazard ratios for risk of death by blood Se level above 50 years of age with *HRG* TT genotype (quartiles).

			Univariate COX Regression			Multivariate COX Regression *		
**Blood Se level μg/L**	**Dead**	**Alive**	**HR**	**95% CI**	***p*-value**	**HR**	**95% CI**	***p*-value**
Q1 <93.96	20(6.9%)	270(93.1%)	2.51	1.14-5.53	0.021	2.40	1.09-5.31	0.029
Q2 93.96-102.81	14(5.2%)	253(94.8%)	1.56	0.67-3.62	0.29	1.52	0.65-3.53	0.33
Q3 102.81-112.40	16(5%)	303(95%)	1.80	0.79-4.08	0.15	1.74	0.76-3.97	0.18
Q4 ref >112.40	9(2.6%)	334(97.4%)	—	—	—	—	—	—

***Adjusted to (smoking , 1st degree relatives, adnexectomy, oral contraception, hormone replacement therapy)**

Table S8. Hazard ratios for any cancer risk by blood Se level below 50 years of age (quartiles).

			Univariate COX Regression			Multivariate COX Regression *		
**Blood Se level μg/L**	**Cases**	**Unaffected**	**HR**	**95% CI**	***p*-value**	**HR**	**95% CI**	***p*-value**
Q1 <93.96	23(7%)	303(93%)	2.017	0.98-4.14	0.05	2.00	0.97-4.12	0.058
Q2 ref 93.96-102.81	11(3.2%)	325(96.8%)						
Q3 102.81-112.40	10(3.4%)	279(96.6%)	1.07	0.45-2.52	0.87	1.07	0.45-2.53	0.86
Q4 >112.40	11(4.6%)	224(95.4%)	1.50	0.65-3.46	0.34	1.51	0.65-3.50	0.33

***Adjusted to (smoking , 1st degree relatives, adnexectomy, oral contraception, hormone replacement therapy)**

Table S9. Hazard ratios for any cancer risk by blood Se level below 50 years of age with *HRG* nonTT genotype (quartiles).

			Univariate COX Regression			Multivariate COX Regression *		
**Blood Se level** μg/L	**Cases**	**Unaffected**	**HR**	**95% CI**	***p*-value**	**HR**	**95% CI**	***p*-value**
Q1 ref<br><93.96	2(2.4%)	81(97.6%)						
Q2<br>93.96-102.81	6(6.4%)	87(93.6%)	2.95	0.59-14.66	0.18	2.85	0.57-14.30	0.20
Q3<br>102.81-112.40	2(2.8%)	68(97.2%)	1.24	0.17-8.85	0.82	1.30	0.18-9.35	0.79
Q4<br>>112.40	2(4.1%)	46(95.9%)	2.00	0.28-14.27	0.48	1.88	0.26-13.58	0.53

***Adjusted to (smoking , 1st degree relatives, adnexectomy, oral contraception, hormone replacement therapy)**

Table S10. Hazard ratios for risk of death by blood Se level below 50 years of age (quartiles).

			Univariate COX Regression			Multivariate COX Regression *		
**Blood Se level** μg/L	**Dead**	**Alive**	**HR**	**95% CI**	***p*-value**	**HR**	**95% CI**	***p*-value**
Q1<br><93.96	2(0.8%)	324(99.2%)	1.30	0.11-14.45	0.82	1.08	0.09-12.16	0.94
Q2<br>93.96-102.81	3(0.8%)	333(99.4%)	2.01	0.20-19.37	0.54	1.80	0.18-17.49	0.61
Q3<br>102.81-112.40	4(1.3%)	285(98.7%)	3.13	0.35-28.06	0.30	2.88	0.32-25.95	0.34
Q4 ref<br>>112.40	1(0.4%)	234(99.6%)						

***Adjusted to (smoking , 1st degree relatives, adnexectomy, oral contraception, hormone replacement therapy)**

Table S11. Hazard ratios for risk of death by blood Se level below 50 years of age with *HRG* TT genotype (quartiles).

			Univariate COX Regression			Multivariate COX Regression *		
**Blood Se level** μg/L	**Dead**	**Alive**	**HR**	**95% CI**	***p*-value**	**HR**	**95% CI**	***p*-value**
Q1 <93.96	2(0.8%)	241(99.2%)	1.42	0.12-15.74	0.77	1.13	0.10-12.84	0.92
Q2 93.96-102.81	3(1.2%)	240(98.8%)	2.20	0.23-21.26	0.49	1.95	0.20-19.03	0.56
Q3 102.81-112.40	4(1.8%)	215(98.2%)	3.40	0.38-30.44	0.27	3.13	0.34-28.32	0.31
Q4 ref >112.40	1(0.5%)	186(99.5%)						

***Adjusted to (smoking , 1st degree relatives, adnexectomy, oral contraception, hormone replacement therapy)**

Table S12. Hazard ratios for risk of death by blood Se level below 50 years of age with *HRG* nonTT genotype (quartiles).

			Univariate COX Regression			Multivariate COX Regression *		
**Blood Se level** μg/L	**Dead**	**Alive**	**HR**	**95% CI**	***p*-value**	**HR**	**95% CI**	***p*-value**
Q1 <93.96	0	83						
Q2 93.96-102.81	0	93						
Q3 102.81-112.40	0	70						
Q4 >112.40	0	48						

***Adjusted to (smoking , 1st degree relatives, adnexectomy, oral contraception, hormone replacement therapy)**
